# Severe Influenza-associated Respiratory Infection in High HIV Prevalence Setting, South Africa, 2009–2011

**DOI:** 10.3201/eid1911.130546

**Published:** 2013-11

**Authors:** Cheryl Cohen, Jocelyn Moyes, Stefano Tempia, Michelle Groom, Sibongile Walaza, Marthi Pretorius, Halima Dawood, Meera Chhagan, Summaya Haffejee, Ebrahim Variava, Kathleen Kahn, Akhona Tshangela, Anne von Gottberg, Nicole Wolter, Adam L. Cohen, Babatyi Kgokong, Marietjie Venter, Shabir A. Madhi

**Affiliations:** National Health Laboratory Service, Johannesburg, South Africa (C. Cohen, J. Moyes, S. Walaza, M. Pretorius, A. Tshangela, A. von Gottberg, N. Wolter, B. Kgokong, M. Venter, S.A. Madhi);; University of the Witwatersrand, Johannesburg (C. Cohen, J. Moyes, M. Groom, E. Variava, K. Kahn, A. von Gottberg, N. Wolter, S. A. Madhi);; Centers for Disease Control and Prevention, Atlanta, Georgia, USA (S. Tempia, A.L. Cohen);; Centers for Disease Control and Prevention–South Africa, Pretoria, South Africa (S. Tempia, A. L. Cohen);; University of KwaZulu-Natal, Durban, South Africa (H. Dawood, M. Chhagan, S. Haffejee);; Pietermaritzburg Metropolitan Hospital, Pietermaritzburg, South Africa (H. Dawood);; Klerksdorp Tshepong Hospital, Klerksdorp, South Africa (E. Variava);; Umeå University, Umeå, Sweden (K. Kahn); INDEPTH Network, Accra, Ghana (K. Kahn);; University of Pretoria, Pretoria (M. Venter)

**Keywords:** influenza, HIV, AIDS, adults, children, pneumonia, pneumococcal, South Africa, viruses, vaccination, lower respiratory tract infection, respiratory infections, co-infection, bacteria, pneumoccocus

## Abstract

Data on influenza epidemiology in HIV-infected persons are limited, particularly for sub-Saharan Africa, where HIV infection is widespread. We tested respiratory and blood samples from patients with acute lower respiratory tract infections hospitalized in South Africa during 2009–2011 for viral and pneumococcal infections. Influenza was identified in 9% (1,056/11,925) of patients enrolled; among influenza case-patients, 358 (44%) of the 819 who were tested were infected with HIV. Influenza-associated acute lower respiratory tract infection incidence was 4–8 times greater for HIV-infected (186–228/100,000) than for HIV-uninfected persons (26–54/100,000). Furthermore, multivariable analysis showed HIV-infected patients were more likely to have pneumococcal co-infection; to be infected with influenza type B compared with type A; to be hospitalized for 2–7 days or >7 days; and to die from their illness. These findings indicate that HIV-infected persons are at greater risk for severe illnesses related to influenza and thus should be prioritized for influenza vaccination.

Knowledge is limited about influenza virus–associated illness and death in persons infected with HIV type 1, particularly in sub-Saharan Africa (*1*,*2*). In 2009, South Africa had ≈5 million HIV-infected persons, and HIV prevalence among pregnant women was 29% (*3*,*4*). Influenza virus circulates seasonally in South Africa, during the Southern Hemisphere winter (*5*).

Studies from the United States suggest that, in the absence of highly active antiretroviral therapy (HAART), HIV-infected adults have an increased risk of seasonal influenza hospitalization (*1*), death (*6*), and prolonged illness compared with the general population. This risk decreased following the widespread introduction of HAART (*6*,*7*). In 2011, ≈52% of eligible HIV-infected adults in South Africa were receiving HAART (*8*), and HAART-naive HIV-infected children had an 8-fold greater risk for influenza-associated pneumonia hospitalization and a trend toward a higher case-fatality rate (CFR) (8% vs. 2% in HIV-uninfected children) (*2*,*9*). Adults in South Africa with AIDS had similar influenza-associated death rates to those for adults in the United States with AIDS in the pre-HAART era (*6*). In Kenya, HIV-infected adults were at increased risk for influenza-associated pneumonia hospitalization compared with HIV-uninfected adults (*10*,*11*).

Data from low HIV prevalence countries where most persons evaluated had access to HAART and influenza antivirals suggested that HIV-infected persons were more likely to be hospitalized for influenza A(H1N1)pdm09 compared with the general population, but rates of intensive care and death did not differ (*1*,*12*). Nevertheless, high HIV prevalence (53%) was observed among patients who died with confirmed influenza A(H1N1)pdm09 in South Africa (*13*). We investigated the incidence of hospitalization for influenza-associated acute lower respiratory tract infection (LRTI) and the clinical course of illness in persons with and without HIV infection in South Africa. 

## Methods

### Surveillance Program

Beginning in February 2009, active, prospective, hospital-based surveillance (the Severe Acute Respiratory Illness program) was implemented in 3 of the 9 provinces of South Africa: Chris Hani-Baragwanath Hospital (CHBH) in an urban area of Gauteng Province; Edendale Hospital in a peri-urban area of KwaZulu-Natal Province; and Matikwana and Mapulaneng Hospitals in a rural area of Mpumalanga Province. In June 2010, an additional surveillance site was introduced at Klerksdorp and Tshepong Hospitals in a peri-urban area of the Northwest Province (Technical Appendix Figure 1).

### Case Definition

A case of acute LRTI was defined as a hospitalized person who had illness onset within 7 days of admission and who met age-specific clinical inclusion criteria. We included children ages 2 days through <3 months who had physician-diagnosed sepsis or acute LRTI, children ages 3 months through <5 years with physician-diagnosed LRTI (e.g., bronchitis, bronchiolitis, pneumonia, pleural effusion), and persons >5 years of age who met the World Health Organization (WHO) case definition for severe acute respiratory infection (*14*): sudden onset of fever (>38°C) or reported fever, cough or sore throat, and shortness of breath or difficulty breathing.

### Study Procedures

All patients admitted during Monday through Friday were eligible, except for adult patients at CHBH, where enrollment was limited to 2 of every 5 working days (selected days varied systematically) per week because of large patient numbers and limited resources. The overall numbers of persons admissions, cases meeting study definitions, and persons enrolled were recorded. Study staff completed case report forms until discharge and collected nasopharyngeal and throat swabs from patients >5 years of age or nasopharyngeal aspirates from patients <5 years of age and blood specimens from consenting patients. Hospital and intensive care unit admission and collection of specimens for bacterial culture, tuberculosis testing, and CD4+ T-cell counts were performed according to attending physician discretion.

### Laboratory Methods

Respiratory specimens were transported in viral transport medium at 4–8°C to the National Institute for Communicable Diseases within 72 hours of collection. Respiratory specimens were tested by multiplex real-time reverse transcription PCR for 10 respiratory viruses as described and included influenza A and B viruses (*15*). Influenza-positive specimens were subtyped by using real-time reverse transcription PCR (*16*). *Streptococcus pneumoniae* was identified by quantitative real-time PCR detecting the *lytA* gene from whole-blood specimens (*17*).

### Definitions

Underlying medical conditions were defined as asthma, other chronic lung disease, chronic heart disease, liver disease, renal disease, diabetes mellitus, immunocompromising conditions excluding HIV infection, neurologic disease, or pregnancy. These conditions were considered absent if indicated as such in medical records or if there was no direct reference to the condition. Invasive isolates were defined as bacterial pathogens, excluding likely contaminants, isolated from blood, cerebrospinal fluid, or another sterile site from a specimen taken within 48 hours of hospitalization. Current tuberculosis was defined patients who had laboratory-confirmed diagnosis of tuberculosis or who were receiving or initiated on anti-tuberculosis treatment during the current admission.

### Evaluation of HIV Serostatus

HIV infection status was determined from results of testing undertaken as part of standard-of-care or through anonymized linked dried blood spot specimen testing, by HIV PCR for children <18 months of age and by ELISA for persons >18 months of age (*18*). CD4+ T-cell counts were determined by flow cytometry (*19*). Patients were categorized into 2 immunosuppression categories (*1*): mild immunosuppression (CD4+ T-lymphocytes >200/mm^3^ or equivalent age-appropriate CD4+ percentage for children <5 years of age), or (*2*) severe immunosuppression (CD4+ T-lymphocytes <200/mm^3^ or equivalent age-appropriate CD4+ percentage for children <5 years of age) (*20*).

### Calculation of Incidence

Calculation of incidence was conducted at CHBH, the only site for which population denominator data were available. This hospital is the only public hospital serving a community of ≈1.3 million black African persons in 2011, of whom ≈10% have private medical insurance (*21*). Most (>80%) uninsured persons and ≈10% of insured persons seek care at public hospitals; consequently, most persons requiring hospitalization from this community are admitted to CHBH. We estimated the incidence of influenza hospitalizations per 100,000 persons by using the number of acute LRTI hospitalizations for which the patient tested positive for influenza virus, adjusting for nonenrollment (i.e., refusal to participate, nonenrollment during weekends, nonenrollment in 3 of 5 adult wards) by age groups and HIV status divided by the midyear total population estimates (*22*) for each year, multiplied by 100,000. HIV prevalence in the study population was estimated from the projections of the Actuarial Society of South Africa AIDS and Demographic model (*3*). We assumed that the HIV prevalence by age group and influenza subtype among patients not tested for HIV was the same as that among those tested. For 14 patients for whom influenza A virus subtyping was not performed, we imputed the influenza subtype on the basis of date of specimen collection and circulating influenza subtypes.

CIs for incidence estimates were calculated by using Poisson distribution. Age-specific and overall age-adjusted risk of influenza hospitalization in HIV-infected and -uninfected persons was determined by using log-binomial regression. To explore the possible effect of missing data on estimates of HIV-specific incidence, a sensitivity analysis was conducted in which all cases not tested for HIV were assumed to be HIV uninfected.

### Analysis of Risk Factors for HIV-Positive Serostatus

Univariate and multivariable analyses were performed in Stata version 9 (StataCorp LP, College Station, TX, USA). Multivariable logistic regression models were evaluated starting with all variables that were significant at p<0.1 on univariate analysis and dropping nonsignificant factors with stepwise backward selection. All 2-way interactions were evaluated. Two-sided p values <0.05 were considered significant. For each univariate analysis, we used all available case information. For the multivariable model, patients with missing data for included variables were dropped. Age group, duration of hospitalization, and year were defined as categorical variables in multiple levels. All other variables were defined as the presence or absence of the attribute, excluding missing data. To explore possible bias, patients tested for HIV were compared with those not tested.

## Results

### Demographics, Clinical Characteristics, and Seasonality of Influenza-associated Acute LRTI

During February 2009–December 2011, a total of 14,725 persons who fulfilled the LRTI case definition were approached for study enrollment; 2,562 (17%) were not enrolled. The most common reasons for nonenrollment were study refusal (n = 779, 30%), unavailable legal guardian (n = 758, 30%), and patients being confused or too ill to consent (n = 242, 9%). Of 12,163 patients enrolled, 11,925 (98%) were tested for influenza; 1,056 (9%) had positive results (Technical Appendix Figure 2). The influenza detection rate varied by age group: 7% (266/4,046) for those <1 year of age, 11% (252/2,292) for those 1–4 years of age, 12% (111/934) for those 5–24 years of age, 9% (270/2,930) for those 25–44 years of age, 9% (119/1,395) for those 45–64 years of age, and 12% (38/328) for those >65 years of age (p<0.001). The overall influenza detection rate was similar among HIV-infected (358/4,208 [9%]) and HIV-uninfected (461/4,473 [10%]) persons (p = 0.163). Most patients (8,961/12,163 [74%]) were enrolled at CHBH.

**Figure 1:**
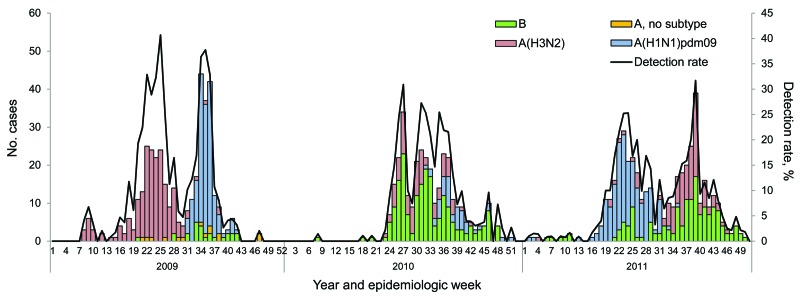
Number of patients testing influenza positive by subtype and influenza detection rate by epidemiologic week and year among patients with hospitalized pneumonia at 4 sentinel surveillance sites, South Africa, 2009–2011.

In 2009, influenza circulation in South Africa was biphasic, with a peak of influenza A(H3N2) infections (190/386 [49%] of annual cases), followed by a second peak of influenza A(H1N1)pdm2009 infections (158/386 [41%] of annual cases). In 2010, influenza B was the predominant subtype (172/289 [60%] of annual cases). In 2011, there were again 2 influenza peaks; influenza A(H1N1)pdm09 predominated (152/381 [40%] of annual cases) initially, followed by influenza B and A(H3N2) (129/381 [34%] and 100/381 [26%] of annual cases, respectively) (Figure 1).

Of the 1,056 patients who had positive test results for influenza, 819 (78%) had an available HIV infection status result (597 [73%] by anonymized HIV testing; 83 [10%] tested by ward clinicians; 139 [17%] by anonymous and clinician testing) (Technical Appendix Figure 2). Age-specific HIV prevalence findings were not substantially different when only patients tested through anonymized unlinked testing were included (data not shown). The proportion of influenza-positive patients with available HIV results increased during the study period, from 62% (239/386) in 2009 to 89% (339/381) in 2011 (p<0.001), and increased with increasing age, from 65% (335/518) among children <5 years of age to 90% (484/538) among persons >5 years of age (p<0.001). When we compared patients tested for HIV to those not tested for HIV, controlling for year of test and age group, no differences in patient epidemiologic characteristics or CFRs were seen (data not shown). The proportion of patients tested for HIV and the HIV prevalence among tested patients did not differ between surveillance sites (data not shown). The overall HIV prevalence among influenza-positive case-patients was 44% (358/819) and varied by age group: 10% (16/164) for those <1 year of age, 17% (29/171) for those 1–4 years of age, 46% (38/82) for those 5–24 years of age, 84% (212/251) for those 25–44 years of age, 54% (61/113) for those 45–64 years of age, and 5% (2/38) for those >65 years of age (p<0.001).

Among patients who had positive influenza test results, 10% (106/1,056) had tuberculosis co-infection, 7% (63/889) had pneumococcal co-infection, and 7% (78/1,056) had another underlying medical condition. Among 106 patients classified as having tuberculosis, only 31 (29%) were laboratory confirmed. Three pregnant women identified in this surveillance tested influenza positive; all were HIV infected. No influenza-positive patient reported receiving influenza vaccine or oseltamivir treatment. Forty-eight HIV-infected and 116 HIV-uninfected patients with influenza had sterile site specimens submitted for bacterial culture; test results were positive for 3 HIV-infected patients (2 *S. pneumoniae* and 1 *Haemophilus influenzae*) and 2 HIV-uninfected patients (1 *Neisseria meningitidis* and 1 *S. pneumoniae*).

### Incidence of Influenza Hospitalization in HIV-Infected and -Uninfected Patients

**Figure 2:**
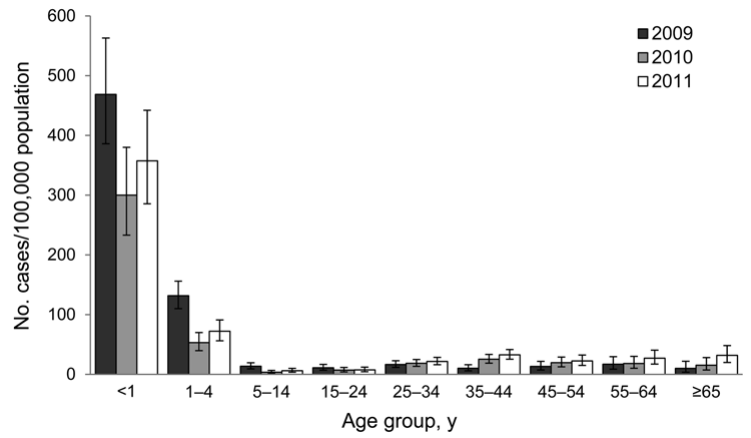
Incidence of laboratory-confirmed influenza-associated lower respiratory tract infection hospitalization, per 100,000 population, by year and age group, at Chris Hani-Baragwanath Hospital, South Africa, 2009–2011. Error bars indicate 95% CIs.

The incidence of hospitalization for influenza-associated acute LRTI among patients at CHBH was highest for patients ages 0–4 years in all study years and for all influenza subtypes, with the highest incidence for those <1 year of age (Table 1; Figure 2). Smaller peaks in incidence were observed in the adult (25–54 years) and elderly (>65 years) age groups each year (Figure 2). HIV-infected persons experienced a 4–8 times greater incidence of influenza-associated acute LRTI (age-adjusted relative risk [aRR] 4.2 [95% CI 3.6–4.8] in 2009, aRR 7.5 [95% CI 6.4–8.8] in 2010, and aRR 5.5 [4.7–6.3] in 2011) (Table 1). The incidence of hospitalization among HIV-infected persons compared with HIV-uninfected persons was 3–5 times greater for influenza A(H3N2) (aRR 3.3 [95% CI 2.7–4.0] in 2009 and aRR 4.9 [3.5–6.5] in 2011), 4–6 times greater for influenza A(H1N1)pdm09 (aRR 4.4 [95% CI 3.6–5.4] in 2009 and 5.6 [95% CI 4.4–7.1] in 2011), and 9 times greater for influenza B [aRR 8.7 (13.2–38.5] in 2010 and 8.7 [4.4–7.2] in 2011) (Technical Appendix Table 1). The relative risk for hospitalization for influenza-associated acute LRTI among HIV-infected persons was elevated in all age groups (generally highest in age group 25–44 years) and for all influenza subtypes; however, this difference was not statistically significant for children 0–4 years of age in some analyses (Table 1; Technical Appendix Table). On sensitivity analysis, assuming that all patients not tested for HIV were HIV negative, the trend toward a higher incidence of influenza in HIV-infected persons remained in all age groups and subtypes except among those 0–4 years of age, the group that had the lowest proportion of patients tested for HIV.

**Table 1:** Incidence of laboratory-confirmed influenza-associated lower respiratory tract infection hospitalizations per 100,000 population by year and HIV status at Chris Hani-Baragwanath Hospital, South Africa*

Year and patient age range, y	No. HIV-positive/no. tested (%)	% HIV prevalence	Incidence rate (95% CI)				Relative risk (95% CI)	
			All patients	HIV infected patients	HIV uninfected patients		HIV infected vs. uninfected	Sensitivity analysis†
2009								
0–4	103/188 (55)	11	336 (304–370)	766 (553–1,021)	314 (284–349)		**2.4 (1.7–3.3)**	1.3 (0.8–1.9)
5–24	18/29 (62)	39	27 (23–33)	194 (142–261)	17 (14–22)		**11.0 (7.4–16.1)**	**5.5 (3.5–8.5)**
25–44	41/44 (93)	88	59 (52–67)	198 (173–227)	9 (7–14)		**20.3 (13.8–31.3)**	**12.7 (9–17.9)**
≥45	27/27 (100)	41	67 (57–78)	260 (201–331)	44 (36–54)		**5.9 (4.2–8.2)**	**5.9 (4.2–8.2)**
Total	189/288 (66)	34	78 (73–83)	228 (206–254)	54 (50–60)		**4.2 (3.6–4.8)**‡	**3.3 (2.9–3.8)**‡
2010								
0–4	54/84 (64)	9	153 (131–177)	317 (187–514)	145 (124–170)		**2.2 (1.3–3.6)**	1.4 (0.7–2.5)
5–24	15/22 (68)	33	14 (11–18)	89 (57–135)	10 (7–13)		**8.8 (5.2–15.2)**	**5.2 (2.8–9.5)**
25–44	73/78 (94)	89	60 (53–68)	203 (178–231)	9 (6–13)		**22.9 (15.4–34.7)**	**14.1 (10.1–19.8)**
≥45	38–39 (97)	55	47 (40–56)	243 (191–307)	24 (18–31)		**10.3 (7.2–14.8)**	**9.7 (6.8–13.9)**
Total	180/223 (81)	53	49 (45–53)	197 (176–219)	26 (23–29)		**7.5 (6.4–8.8)**‡	**6.2 (5.3–7.3)**‡
2011								
0–4	81/96 (84)	6	186 (162–212)	273 (151–463)	182 (159–209)		1.5 (0.8–2.6)	1.3 (0.6–2.3)
5–24	13/13 (100)	46	8 (6–11)	71 (42–111)	5 (3–7)		**15.4 (7.7–30.3)**	**15.4 (7.7–30.3)**
25–44	88/89 (99)	80	68 (61–76)	206 (180–234)	19 (15–24)		**10.9 (8.2–14.7)**	**10.4 (7.9–14)**
≥45	42/43 (98)	38	56 (48–65)	192 (146–247)	39 (32–48)		**4.9 (3.5–6.9)**	**4.8 (3.4–6.7)**
Total	224/241 (93)	43	54 (50–58)	186 (167–207)	34 (31–37)		**5.5 (4.7–6.3)**‡	**5.3 (4.6–6.2)**‡

*Boldface indicates significance. †Assuming that all patients not tested for HIV are HIV negative. ‡Age-adjusted.

### Characteristics of HIV-Infected Patients and Factors Associated with HIV Infection among Influenza Virus–Positive Patients

Among influenza virus–positive case-patients, the CFR was 4 times greater for HIV-infected (19/356, 5%) than for HIV-uninfected (6/461, 1%) persons (p = 0.002). In each age group except for the elderly, CFRs were significantly higher for HIV-infected compared with HIV-uninfected persons: 7% (36/509) vs. 1% (34/3,630) for ages 0–4 years (relative risk [RR] 7.6, 95% CI 4.7–12.1); 6% (28/433) vs. 1% (3/298) for ages 5–24 years (RR 6.4, 95% CI 2.0–21.1); 7% (164/2,381) vs. 3% (8/308) for ages 25–44 years (RR 2.7, 95% CI 1.3–5.4); 12% (100/833) vs. 7% (34/456) for ages 45–64 years (RR 1.6, 95% CI 1.1–2.4); and 4% (2/50) vs. 9% (23/246) for age >65 years (RR 0.4, 95% CI 0.1–1.8).

Results from multivariable analysis indicate that, among patients with influenza-associated hospitalization, those with HIV infection (compared with those without HIV infection) were more likely to be age group 5–24 years (odds ratio [OR] 4.4, 95% CI 2.4–8.2), 25–44 years (OR 24.2, 95% CI 14.1–41.7), or 45–64 years (OR 6.2, 95% CI 3.4–11.3); female sex (OR 1.9, 95% CI 1.2–2.8); black African race (OR 4.0, 95% CI 1.1–14.6); co-infected with pneumococcus (OR 2.3, 95% CI 1.0–5.0); infected with influenza type B (vs. type A) (OR 1.6 95% CI 1.0–2.4); hospitalized for 2–7 days (OR 2.8 95% CI 1.5–5.5) or >7 days (OR 4.5, 95% CI 2.1–9.5); and more likely to die (OR 3.9, 95% CI 1.1–14.1) (Table 2). In contrast, those with HIV infection were less likely than those without HIV infection to have underlying medical conditions other than HIV (OR 0.4, 95% CI 0.2–0.8).

**Table 2:** Comparison of the clinical and epidemiologic characteristics of HIV-infected and uninfected patients hospitalized with influenza-associated acute LRTI at 4 sentinel surveillance sites, South Africa 2009–2011*

Characteristic	HIV-infected patients†	HIV-uninfected patients†	Univariate analysis			Multivariable analysis	
			OR (95% CI)	p value		OR (95% CI)	p value
Patient demographics							
Age group, y				<0.001			<0.001
<5	45/358 (13)	290/461 (63)	Referent			Referent	
5–24	38/358 (11)	44/461 (10)	5.6 (3.3–9.5)			4.4 (2.4–8.2)	
25–44	212/358 (59)	39/461 (8)	35.0 (22.0–55.7)			24.2 (14.1–41.7)	
45–64	61/358 (17)	52/461 (11)	7.6 (4.7–12.3)			6.2 (3.4–11.3)	
>65	2/358 (1)	36/461 (8)	0.4 (0.1–1.5)			0.2 (0.04–0.9)	
Female sex	253/358 (71)	224/461 (49)	2.5 (1.9–3.4)	<0.001		1.9 (1.2–2.8)	0.003
Black African race	353/358 (99)	445/460 (97)	2.4 (0.9–6.6)	0.096		4.0 (1.1–14.6)	0.036
Year of hospitalization				0.002			
2009	88/358 (25)	151/461 (33)	Referent				
2010	127/358 (35)	114/461 (25)	1.9 (1.3–2.8)				
2011	143/358 (40)	196/461 (43)	1.3 (0.9–1.8)				
Co-infections and underlying medical conditions							
Underlying condition excluding tuberculosis and HIV‡	25/358 (7)	47/461 (10)	0.7 (0.4–1.1)	0.109		0.4 (0.2–0.8)	0.008
Smoking§	32/299 (11)	24/151 (16)	0.6 (0.4–1.1)	0.117			
Consumed alcohol§	28/299 (9)	26/151 (17)	0.5 (0.3–0.9)	0.017			
Underlying tuberculosis	60/357 (17)	19/461 (4)	4.7 (2.7–8.0)	<0.001			
Pneumococcal co-infection on PCR¶	37/345 (11)	17/389 (4)	2.7 (1.5–5.0)	<0.001		2.3 (1.0–5.0)	0.043
Viral respiratory co-infection#	82/358 (23)	152/456 (33)	0.6 (0.4–0.8)	0.001			
Influenza type B (vs. A)	148/358 (41)	133/461 (29)	1.7 (1.3–2.3)	<0.001		1.6 (1.0–2.4)	0.035
Received >2 doses of pneumococcal conjugate vaccine**	3/39 (8)	53/242 (22)	0.3 (0.1–1.0)	0.051			
Clinical findings and treatment course							
Symptoms >2 d before admission	296/358 (83)	295/461 (64)	2.7 (1.9–3.7)	<0.001			
Admission to intensive care	0/357 (0)	6/461 (1)	Undefined	0.031			
Mechanical ventilation	2/357 (1)	4/461 (1)	0.6 (0.1–3.5)	0.612			
Oxygen required	142/357 (40)	141/461 (31)	1.5 (1.1–2.0)	0.006			
Antimicrobial drugs prescribed at admission	351/358 (98)	438/460 (95)	2.5 (1.1–6.0)	0.036			
Duration of hospitalization, d				<0.001			<0.001
<2	20/352 (6)	149/460 (32)	Referent			Referent	
2–7	217/352 (62)	241/460 (52)	6.7 (4.1–11.1)			2.8 (1.5–5.5)	
>7	115/352 (33)	70/460 (15)	12.2 (7.0–21.3)			4.5 (2.1–9.5)	
Median duration of hospitalization, d (range)	6 (4–8)	3 (1–6)	1.1 (1.05–1.13)	<0.001			
Case-fatality rate	19/356 (5)	6/461 (1)	4.3 (1.7–10.8)	0.002		3.9 (1.1–14.1)	0.038

*ORs and p values are shown for all variables included in the multivariable model. LRTI, lower respiratory tract infection; OR, odds ratio. †Values are no. patients/total no. in category (%) except as indicated. Some data are missing or were not recorded. ‡Asthma, other chronic lung disease, chronic heart disease (valvular heart disease, coronary artery disease, or heart failure excluding hypertension), liver disease (cirrhosis or liver failure), renal disease (nephrotic syndrome, chronic renal failure), diabetes mellitis, immunocompromising conditions excluding HIV infection (organ transplant, immunosuppressive therapy, immunoglobulin deficiency, malignancy), neurologic disease (cerebrovascular accident, spinal cord injury, seizures, neuromuscular conditions) or pregnancy. Coexisting illnesses were considered absent in cases for which the medical records stated that the patient had no underlying medical condition or when there was no direct reference to that condition. §Question asked of patients >12 y of age only. ¶Three additional cases of *Streptococcus pneumoniae* on blood culture not included #Co-infection with influenza and >1 of the following: parainfluenza virus 1, 2, or 3; respiratory syncytial virus; enterovirus; human metapneumovirus; adenovirus; rhinovirus. **Verified only for children <5 y of age.

A total of 118 (33%) HIV-infected patients had available CD4+ T-cell count data; 7 were <5 years of age. Most (60%, 70/118) had severe immunosuppression (CD4+ T cell counts <200/mm^3^ or age-specific equivalent). CFRs were not significantly different between patients with (13%, 9/70) and without (6%, 3/47; p = 0.258) severe immunosuppression, although numbers were small. The duration of hospitalization was longer for those with severe immunosuppression (median 7 days, interquartile range 2–11 days) than for those without (median 5 days, interquartile range 1–7 days; p = 0.02). Of those with available data, 51% (111/218) reported currently receiving HAART and 25% (60/241) reported receiving prophylaxis with trimethoprim/sulfamethoxazole. CFRs were similar for patients receiving (7/113, 6%) and not receiving (8/107, 7%) HAART (p = 0.706).

## Discussion

We have shown that HIV-infected persons experienced elevated incidence of hospitalization, prolonged hospitalization, and increased risk of in-hospital death resulting from influenza. In contrast to most other countries (*12*), HIV infection (>40%) was the most common underlying risk factor for influenza-associated LRTI hospitalization in South Africa. This factor resulted in a W-shaped age-distribution of influenza hospitalizations, with peaks among young children and the elderly and an additional peak among young adults associated with HIV infection. These findings highlight the need to target HIV-infected persons for influenza vaccination.

Bacterial co-infections may have contributed to some of the influenza-associated LRTI hospitalizations and deaths in the HIV-infected group, among whom we observed an elevated risk of pneumococcal co-infection. An elevated risk of hospitalization for invasive pneumococcal disease has been documented in HIV-infected persons (*23*), and a synergistic relationship exists between influenza and pneumococcus (*24*,*25*). Whereas real-time PCR is more sensitive than blood culture for diagnosing pneumococcal pneumonia, additional cases of pneumococcal co-infection may still have been missed (*23*,*26*). Pneumococcal DNA in the blood may reflect occult bacteremia in some persons (*27*,*28*).

HIV-infected persons with influenza-associated acute LRTI were more likely to have underlying tuberculosis, although not all tuberculosis cases were laboratory-confirmed. Tuberculosis was also common in a South African case-series of influenza A(H1N1)pdm09 deaths (*13*). An association between tuberculosis and influenza-associated death has been suggested (*11*,*29*) but warrants further corroboration.

The observed prevalence of underlying medical conditions was lower for HIV-infected (7%) than HIV-uninfected persons (10%) and lower than has been observed in the United States, where 68% of HIV-infected and 74% of hospitalized HIV-uninfected adults had influenza A(H1N1)pdm09 (*12*). This discrepancy could be because our documentation was incomplete or may reflect a true difference in the relative contribution of underlying risk conditions in our setting.

The increased risk for hospitalization for influenza-associated acute LRTI among HIV-infected persons appeared to be greater for influenza B (≈8-fold) than influenza A (≈3–4-fold). Reasons for this are unclear. Influenza B severity is intermediate, falling between those for influenza A(H3N2) and A(H1N1). Bacterial superinfection may contribute to death in patients (particularly adults) with influenza B, and severe and fatal disease due to influenza B has been described in previously healthy persons (*30*).

Influenza vaccination is safe and efficacious in HIV-infected adults in Africa (*31*,*32*), whereas the efficacy among HIV-infected children is unclear (*33*). No patients reported receiving influenza vaccination or antiviral treatment, despite national recommendations for influenza vaccination of risk groups and for antiviral treatment for influenza infection in persons with severe illness or underlying risk conditions (*34*). Influenza vaccine (170,000–1,000,000 doses for a population of ≈50 million each year) and oseltamivir treatment are made available free of charge through the public health sector in South Africa, although challenges in procurement and distribution may limit access. The low uptake of oseltamivir may be because clinicians doubt its effectiveness when patients delay seeking health care; >80% of HIV-infected persons reported symptoms for >48 hours before admission. The effectiveness of antiviral treatment for influenza- associated LRTI hospitalization in settings similar to ours needs to be evaluated. An additional contributing factor to the low use of oseltamivir could be a low index of suspicion for influenza as an etiologic agent in HIV-infected persons with LRTI, because they are also at risk for respiratory disease from other pathogens, such as pneumococcus, *Pneumocystis jirovecii*, and tuberculosis (*12*). Maternal immunization against influenza has been suggested as a strategy to reduce the high rates of influenza infection among infants <6 months of age (*35*), but the effectiveness of this intervention in settings with a high prevalence of maternal HIV infection is unknown. 

Our study has several limitations. The low rate of HIV testing among children may have introduced bias if their characteristics differed from those who were tested. Surveillance programs such as ours may underestimate the true number of deaths because severely ill patients may be less amenable to study inclusion or may die before or shortly after hospital admission. Our estimates of incidence also assumed that all persons in Soweto access care at CHBH hospital. Therefore, our estimates likely represent minimum rates. Nevertheless, the estimates of relative risk by HIV status should be robust, unless patients had differential access to care by HIV-infection status (*12*). Incidence data were derived from a temperate urban area and may not be representative of more subtropical rural areas, but incidence among HIV-uninfected persons was similar to that described for other developing countries (*36*,*37*). This analysis included the years after the introduction of influenza A(H1N1)pdm09, and thus we cannot comment on age-specific influenza incidence before this period. Several studies have suggested that pregnancy is a major risk factor for severe disease and death associated with influenza virus infection (*38*,*39*). Few pregnant women were enrolled in our study; these patients may have been missed because review of admissions to maternity wards was not always consistent. The case definition of physician-diagnosed acute LRTI in children ages 3 months–<5 years relied on subjective clinician assessment and did not include fever as a criterion because acute LRTI may be afebrile and fever reporting may be subjective in this age group. CD4+ cell count data were only available for one third of HIV-infected patients, and CD4+ cell counts among tested patients may have differed from those in untested patients.

In conclusion, we have demonstrated that, in a high HIV-prevalence setting, HIV infection is a major risk factor for influenza hospitalization and severe disease. Further studies are warranted on the effectiveness of influenza vaccine among HIV-infected children and HIV-infected adults with advanced immunosuppression or tuberculosis co-infection. 

Technical Appendix Geographic location of 4 sentinel surveillance sites, outcome for patients with severe influenza-associated lower respiratory tract infection in a high HIV prevalence setting, and incidence rates for of laboratory-confirmed influenza-associated lower respiratory tract infection hospitalizations at Chris Hani-Baragwanath Hospital, South Africa, 2009–2011.
