# Supplementary material for: Severe Influenza-associated Respiratory Infection in High HIV Prevalence Setting, South Africa, 2009–2011
Source: Emerg Infect Dis. 2013 Nov;19(11):1766–74. doi: 10.3201/eid1911.130546 (PMC3837669; doi:10.3201/eid1911.130546)
Supplement: Technical Appendix — Geographic location of 4 sentinel surveillance sites, outcome for patients with severe influenza-associated lower respiratory tract infection in a high HIV prevalence setting, and incidence rates for of laboratory-confirmed influenza-associated lower respiratory tract infection hospitalizations at Chris Hani-Baragwanath Hospital, South Africa, 2009–2011. [file 13-0546-Techapp-s1.pdf]

Article DOI: http://dx.doi.org/10.3201/eid1911.130546

Severe Influenza-Associated Respiratory Infection in High HIV Prevalence Setting, South Africa, 2009–2011

Technical Appendix

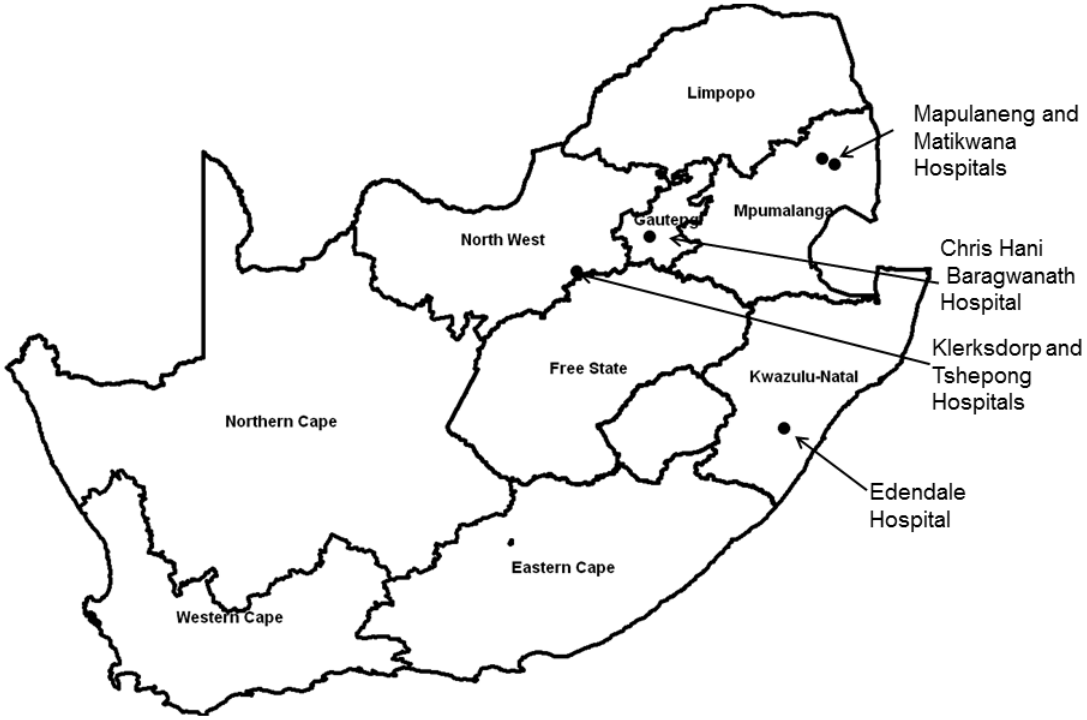

Technical Appendix Figure 1. Geographic location of 4 hospital-based influenza surveillance sites in the 9 provinces of South Africa. The sentinel sites are part of the Severe Acute Respiratory Illness (SARI) program.

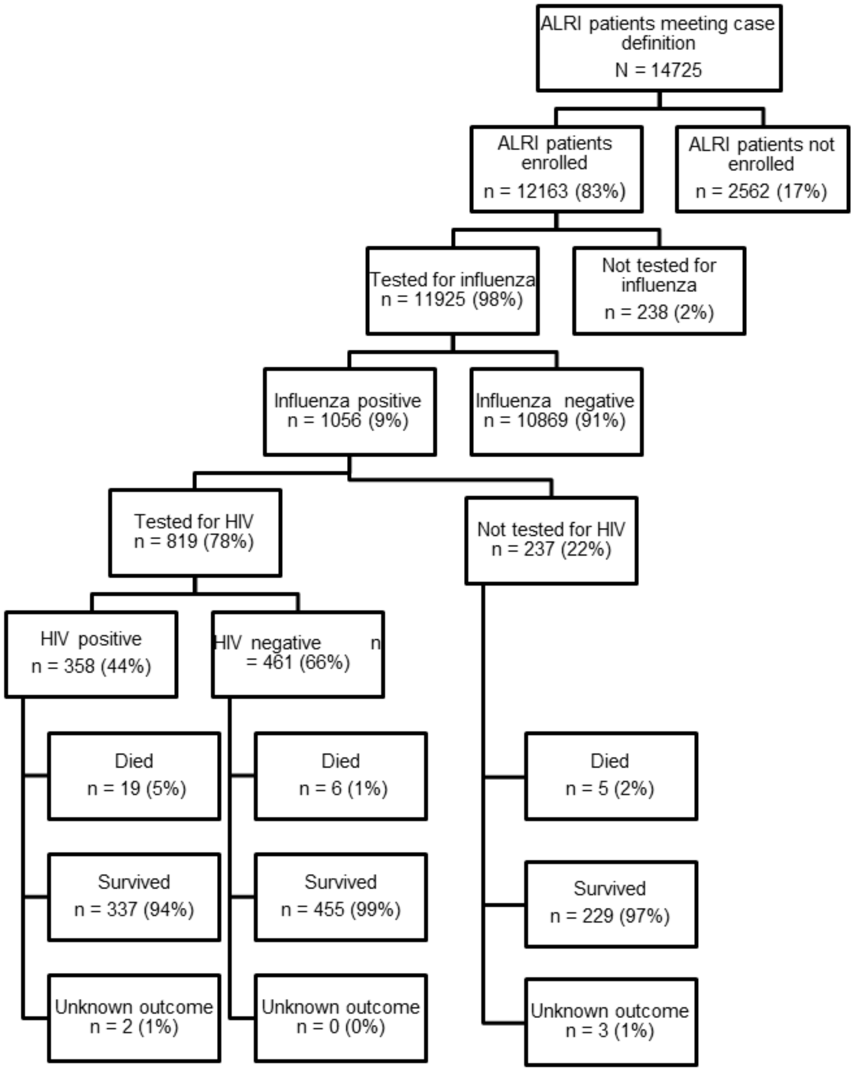

Technical Appendix Figure 2. Outcome for patients meeting the case definition for severe influenza-associated lower respiratory tract infection in a high HIV prevalence setting, South Africa, 2009–2011. ALRI, acute lower respiratory tract infection.

Technical Appendix Table. Incidence rate (IR) of laboratory-confirmed influenza associated lower respiratory tract infection hospitalization per 100,000 population by year, HIV status and influenza type or subtype, Chris Hani-Baragwanath Hospital, South Africa, 2009–2011*

Year	Dominant influenza type/subtype	Age group (years)	% with HIV result	HIV prevalence, %	IR (95% CI) All patients	IR (95% CI) HIV infected	IR (95% CI) HIV uninfected	RR (95% CI) HIV infected vs HIV uninfected	RR (95% CI) HIV infected vs HIV uninfected sensitivity analysis†
2009	A(H3N2)	0–4	51	8	211 (186–238)	375 (238–576)	202 (178–230)	**1.9 (1.2–2.9)**	0.9 (0.4–1.6)
	A(H3N2)	5–24	53	50	14 (11–18)	129 (88–185)	7 (5–11)	**17.3 (10.1–29.8)**	**6.3 (3.3–11.4)**
	A(H3N2)	25–44	87	100	20 (16–25)	77 (62–96)	0 (0–1)‡	**–**	**18.3 (9.6–38.1)**
	A(H3N2)	≥45	100	38	20 (15–26)	71 (42–112)	13 (9–20)	**5.2 (2.7–9.5)**	**5.2 (2.7–9.5)**
	A(H3N2)	Total	57	28	38 (34–41)	94 (80–111)	28 (26–32)	**3.3 (2.7–4.0)**§	**2.2 (1.8–2.8)**§
	A(H1N1)pdm09	0–4	61	11	109 (92–129)	251 (145–428)	101 (84–122)	**2.5 (1.4–4.4)**	1.4 (0.7–2.9)
	A(H1N1)pdm09	5–24	77	30	12 (9–16)	67 (40–112)	9 (6–12)	**7.4 (4–14.3)**	**5.2 (2.5–10.1)**
	A(H1N1)pdm09	25–44	96	86	30 (25–36)	97 (87–119)	5 (3–9)	**16.9 (10.2–30.5)**	**12.7 (7.9–21)**
	A(H1N1)pdm09	≥45	100	31	40 (32–48)	118 (80–169)	30 (23–39)	**3.9 (2.4–6.1)**	**3.9 (2.4–6.1)**
	A(H1N1)pdm09	Total	75	36	34 (30–37)	101 (87–119)	23 (20–26)	**4.4 (3.6–5.4)**§	**3.8 (3.1–4.7)**§
2010	B	0–4	62	6	94 (78–114)	133 (53–272)	93 (76–112)	1.4 (0.6–3)	0.9 (0.2–2.1)
	B	5–24	77	30	8 (6–11)	47 (24–82)	6 (4–9)	**7.6 (3.5–15.3)**	**5.3 (2.1–11)**
	B	25–44	96	92	37 (32–44)	131 (111–154)	4 (2–7)	**30.3 (17.5–56.9)**	**20.2 (12.7–34.4)**
	B	≥45	96	73	28 (22–35)	189 (143–246)	9 (5–13)	**22.1 (13.2–38.5)**	**19 (11.5–32.7)**
	B	Total	81	58	30 (27–33)	129 (113–147)	15 (13–17)	**8.7 (13.2–38.5)**§	**7.5 (6.1–9.2)**§
2011	A(H3N2)	0–4	65	0	45 (34–58)	0 (0–72)**	47 (35–61)	**0 (0–1.6)**	**0 (0–1.6)**
	A(H3N2)	5–24	25	100	1 (0–2)	12 (2–34)	–	–	**–**
	A(H3N2)	25–44	51	84	15 (11–18)	47 (35–61)	3 (1–6)	**15 (7.7–33.5)**	**15 (7.7–33.5)**
	A(H3N2)	≥45	94	33	19 (15–25)	59 (34–91)	15 (10–20)	**4 (2.1–7)**	**4 (2.1–7)**
	A(H3N2)	Total	63	40	13 (11–15)	42 (34–53)	9 (7–11)	**4.9 (3.5–6.5)**§	**4.9 (3.5–6.5)**§
	A(H1N1)pdm09	0–4	52	8	85 (70–104)	160 (68–311)	82 (66–100)	2.0 (0.8–3.9)	1.7 (0.7–3.6)
	A(H1N1)pdm09	5–24	67	38	5 (3–7)	35 (16–67)	3 (2–5)	**10.8 (4.2–26.3)**	**10.8 (4.2–26.2)**
	A(H1N1)pdm09	25–44	26	84	25 (208–303)	81 (66–100)	5 (3–9)	**15.2 (8.9–26.1)**	**12.7 (7.9–22.2)**
	A(H1N1)pdm09	≥45	19	46	16 (11–21)	64 (39–99)	10 (6–14)	**6.7 (3.5–12.6)**	**5.7 (2.9–10.5)**
	A(H1N1)pdm09	Total	33	43	21 (19–24)	74 (62–88)	13 (11–15)	**5.6 (4.4–7.1)**§	**5.2 (4.1–6.6)**§
	B	0–4	81	8	60 (47–75)	133 (43–258)	57 (45–74)	1.4 (0.7–4.7)	1.4 (0.5–4.1)
	B	5–24	100	50	2 (1–4)	23 (9–51)	1 (0–3)	**7.6 (4.8–67.1)**	**7.6 (4.8–67.1)**
	B	25–44	100	73	28 (24–34)	78 (64–97)	10 (7–15)	**30.3 (5.1–11.6)**	**30.3 (5.1–11.6)**
	B	≥45	100	38	21 (16–27)	189 (44–106)	9 (10–20)	**22.1 (2.7–8.3)**	**22.1 (2.7–8.3)**
	B	Total	93	45	20 (18–23)	129 (58–84)	15 (10–15)	**8.7 (4.4–7.2)**§	**8.7 (4.3–7.1)**§

*Significant relative risk values at p<0.05 in boldface. IR, incidence rate; RR, relative risk.
†Assuming that all patients not tested for HIV are HIV negative.
‡One-sided.
§Age-adjusted.
